# Detection of Pathological Voice Samples Using a Text-to-Speech–Inspired Approach With Multiple Model Architectures: Method Development and Comparative Evaluation Study

**DOI:** 10.2196/94285

**Published:** 2026-10-19

**Authors:** Marie Sophie Walter, Muhittin Demir, Mattias Heinrich, Felix Nensa, Stephan Lang, Nadia Sadok, René Hosch

**Affiliations:** 1 Institute of Diagnostic and Interventional Radiology and Neuroradiology, University Hospital Essen, Hufefelandstraße 55, Essen, 45147, Germany, 49 20172377817; 2 Institute for Artificial Intelligence in Medicine (IKIM), University Hospital Essen, Essen, Germany; 3 Department of Otorhinolaryngology, Head and Neck Surgery, University Hospital Essen, University Duisburg-Essen, Essen, Germany; 4 University of Lübeck, Institute of Medical Informatics, Lübeck, Germany

**Keywords:** voice disorders, text-to-speech, deep learning, classification, synthetic speech augmentation

## Abstract

**Background:**

Voice disorders are common, yet access to timely specialist assessment is often limited. Automated analysis of short voice recordings may support early screening, but achieving robust performance on heterogeneous clinical datasets remains challenging.

**Objective:**

This study aims to investigate the impact of synthetic speech augmentation on voice disorder recognition and classification using the Saarbrücken Voice Database (SVD) and to develop an adaptive ensemble with learnable weighting and optional sex embedding.

**Methods:**

Sentence recordings from the SVD were used for binary voice disorder recognition (VDR, n=1988) and 4-class voice disorder classification (VDC; n=1199). Two text-to-speech (TTS) models, XTTS and Kartoffelbox, generated 1 synthetic sample for each training instance. Synthetic speech quality was assessed in a blinded listening evaluation with 40 samples per TTS model. Baseline models included support vector machine (SVM), ResNet-18, long short-term memory, and transformer-based architectures, including Hidden-Unit BERT (HuBERT), Wav2Vec 2.0, and WavLM. Ensemble learning and learnable fusion (LF), with and without a sex embedding (LFS), were evaluated using bootstrapped performance estimates and standard classification metrics. Area under the receiver operating characteristic curve (AUC) was reported for VDR.

**Results:**

In the blinded evaluation, Kartoffelbox-generated synthetic samples were less often identified as synthetic than XTTS-generated synthetic samples (10/19, 52.6% vs 17/19, 89.5%). In pooled original and synthetic ratings, Kartoffelbox showed higher pathological-status identification than XTTS for pathological samples (22/29, 75.9% vs 19/29, 65.5%) and nonpathological samples (8/10, 80.0% vs 6/10, 60.0%), as well as higher diagnostic-class assignment (20/39, 51.3% vs 14/39, 35.9%). Among the baseline models, HuBERT showed the most balanced VDR performance (accuracy=0.85, *F*_1_-score=0.84). Kartoffelbox-based augmentation further improved performance, with HuBERT achieving 0.88 sensitivity, 0.87 accuracy, and 0.85 *F*_1_-score. LF showed minor improvements in threshold-dependent metrics (accuracy=0.87, *F*_1_-score=0.86), while LFS further balanced sensitivity and specificity (accuracy=0.88, AUC=0.92). The equal-weight ensemble achieved the highest AUC among ensemble approaches (AUC=0.93). For VDC, HuBERT was the strongest baseline (accuracy=0.72, median *F*_1_-score=0.63), and LF achieved the highest overall fusion performance (accuracy=0.73, *F*_1_-score=0.66), while laryngitis remained one of the more challenging classes.

**Conclusions:**

High-quality synthetic speech augmentation can improve automated voice disorder analysis across heterogeneous clinical data, with effectiveness varying by the TTS model. Multimodel fusion of complementary architectures provided robust performance for screening and multiclass classification, with the strongest approach varying by metric and task. These findings support further evaluation of AI-assisted preassessment in clinical and telemedicine settings.

## Introduction

Voice disorders are a common and clinically relevant condition with a substantial impact on quality of life, occupational performance, and health care use. They encompass a broad spectrum of etiologies, ranging from benign and self-limiting conditions to severe and potentially life-threatening diseases, including laryngeal cancer. Population-based studies in Germany indicate a high prevalence of voice disorders [1,2], with lifetime risk estimates suggesting that more than 30% of individuals experience dysphonia at some point. Professional voice users, such as teachers and singers, are particularly affected. Clinically, voice disorders are typically categorized into functional, organic, and neurological subtypes [3], reflecting heterogeneous etiologies and acoustic manifestations. Functional disorders often arise from vocal misuse or overuse in the absence of structural pathology, whereas organic disorders are associated with morphological changes of the vocal folds, ranging from inflammatory conditions to malignant diseases, including laryngeal cancer, with laryngitis being among the most common diagnoses [2]. Neurological voice disorders may occur secondary to neurodegenerative diseases, such as Parkinson disease or amyotrophic lateral sclerosis, or result from primary neural impairments affecting vocal fold innervation, including recurrent laryngeal nerve paresis [3]. Importantly, acoustic manifestations may overlap across these etiological categories, and similar perceptual voice characteristics can arise from distinct underlying pathophysiological mechanisms [3]. This overlap complicates automated classification of voice disorders and underscores the need for robust machine learning approaches capable of identifying subtle disease-specific patterns.

Early identification and treatment of voice disorders are crucial, as timely intervention has been shown to improve outcomes, shorten symptom duration, and reduce the risk of chronic impairment; importantly, early detection of malignant laryngeal disease enables prompt oncologic management and is associated with significantly improved prognosis and survival [4-6]. However, access to specialized otolaryngology and phoniatrics care remains limited in many health care systems, with long waiting times that frequently delay diagnosis and therapy. These barriers highlight the need for scalable, technology-based screening approaches that can support early detection and referral. In this context, automated analysis of short speech or voice recordings using AI has emerged as a promising digital health solution for detecting and classifying voice disorders [7-10].

Recent studies demonstrate strong performance of AI-based models for pathological voice recognition, particularly when evaluated on curated benchmark datasets. Notably, models trained on the Voice Icar Federico (VOICED) database [11] have achieved very high sensitivity and specificity across a range of architectures [8-10]. For example, Peng et al [8] reported a sensitivity of 0.996 and a specificity of 0.989. However, the VOICED dataset comprises only 208 samples across 4 diagnostic classes, limiting acoustic variability and constraining the generalizability of models to real-world clinical populations. The Saarbrücken Voice Database (SVD) [12], in contrast, includes metadata from 2225 speaker or recording-session entries covering 72 diagnostic categories and therefore better reflects the heterogeneity encountered in clinical practice. This increased diversity, however, also results in greater task complexity and lower reported performance. Using the SVD, Verde et al [13] achieved a sensitivity of 0.82 and a specificity of 0.83 with regression-based methods, while Koudounas et al [7] reported an accuracy of 0.91 (SD 0.006) and an area under the curve of 0.91 (SD 0.005) using an ensemble of transformer-based models. A substantial body of voice-disorder research has used sustained vowels because they provide controlled phonatory signals and allow extraction of stable acoustic and glottal features. These studies are highly relevant for pathological voice detection, including work on convolutional neural networks with electroglottographic and speech signals [14] and recurrence plot-based characterization of speech dynamics in hyperfunctional dysphonia, laryngitis, and vocal fold polyp [15]. However, they differ from the present setting, which focuses on standardized sentence recordings and TTS-generated connected speech. Sentence recordings include articulatory, temporal, and prosodic information that may be important for screening applications and introduce greater acoustic variability.

Data scarcity and class imbalance remain major challenges in training robust voice disorder recognition models, particularly for rare diagnostic categories. Recent work suggests that synthetic speech generated via text-to-speech (TTS) models can partially mitigate these limitations by augmenting training data and improving model performance [7]. However, it remains unclear how the choice of TTS model, the quality of synthetic speech, and their interaction with different model architectures influence performance, especially in heterogeneous clinical datasets and beyond binary screening tasks. Previous studies have demonstrated strong performance of transformer-based models for pathological voice recognition and have begun to explore synthetic speech as a strategy for increasing training data. However, it remains unclear whether synthetic speech improves robustness consistently across model architectures, whether the perceptual quality of the TTS system affects downstream performance, and whether gains observed in binary screening transfer to more clinically specific multiclass classification. The present study addresses this gap by jointly evaluating 2 TTS systems; multiple model families; an equal-weight ensemble, learnable fusion; and optional sex-aware fusion on the heterogeneous SVD dataset.

In this study, we address these gaps by systematically investigating synthetic speech augmentation for voice disorder analysis using 2 complementary TTS approaches: XTTS [16], a multilingual TTS model, and Kartoffelbox, a German-only TTS system. We explicitly evaluate the quality of the generated synthetic speech and analyze its impact across multiple model architectures. Building on this, we propose an ensemble framework with learnable weighting that leverages the complementary strengths of heterogeneous models for voice disorder recognition (VDR) in a binary classification setting (pathological vs nonpathological). Motivated by prior evidence of sex-dependent differences in vocal characteristics and model performance, we additionally incorporate a sex embedding into the modeling pipeline. Finally, we extend our evaluation to the more challenging voice disorder classification (VDC) task, which requires predicting specific diagnostic categories, thereby assessing the applicability of the proposed approach to clinically relevant, fine-grained analysis of voice disorders. By jointly evaluating TTS-based augmentation, foundation-model architectures, and clinically informed multimodel fusion within a unified framework, this study provides a comprehensive assessment of automated voice disorder recognition and classification in heterogeneous clinical data.

## Methods

### Ethical Considerations

As this study exclusively used publicly available, deidentified datasets, no additional ethical approval or formal ethics statement was required.

### Dataset Characteristics

This study used the SVD [12], a large, well-established German-language corpus comprising annotated voice recordings from both pathological and nonpathological speakers. Recordings were collected between June 20, 1997, and June 16, 2004, at the Institut für Phonetik at Saarland University and the Phoniatry Section of the Caritas Clinic St. Theresia in Saarbrücken, Germany.

The SVD comprises metadata for 2225 speaker/recording-session entries (mean age 41.7, SD 18.1, range 6‐94 years; n=1163, 52.3% female participants), including 869 entries labeled as nonpathological and 1356 entries labeled as pathological. In the raw database folder available for this study, 57,677 files were present, including 28,515 acoustic signal files (.nsp), 28,515 corresponding electroglottography files (.egg), and 647 remarks files (.txt). The recording protocol comprised a standardized spoken sentence (“Guten Morgen, wie geht es Ihnen”; English: “Good morning, how are you?”) and sustained vowel phonations (“i,” “a,” “u”) produced at neutral, low, and high pitch. Not all recording types were available for every participant. For the present analyses, only standardized sentence recordings were used, with 1988 usable phrase recordings retained after preprocessing and eligibility filtering. An overview of the dataset composition is provided in Figure 1.

Diagnostic-label assignments comprised 71 pathological diagnostic categories and 1 nonpathological category (Table S5 in Multimedia Appendix 1). Individual pathological speakers could carry more than one diagnostic label, so the supplementary class distribution reports diagnostic-label assignments rather than mutually exclusive speaker counts. The original SVD labels comprise a heterogeneous mixture of clinical diagnoses and symptom-based annotations. For VDR, all 71 pathological categories were combined into one pathological class because this task was designed to approximate an initial screening scenario. In such a setting, the clinically relevant first step is to distinguish voices that may require specialist assessment from voices without documented pathology. This binary grouping intentionally does not imply acoustic or etiological equivalence among the included disorders. Rather, it reflects a triage-oriented use case before subsequent clinical examination or a more fine-grained diagnostic classification.

In addition, for the VDC task, analyses were restricted to 4 clinically relevant diagnostic categories (nonpathological, hyperfunctional dysphonia, laryngitis, and recurrent laryngeal nerve paresis) that were sufficiently represented within the dataset to enable robust supervised classification. Furthermore, only sentence recordings were used in both the VDR and VDC experiments.

For both the VDR and VDC experiments, only standardized sentence recordings were analyzed. However, during preprocessing, sentence recordings were retained only when the corresponding recording ID could be matched to a paired sustained-vowel recording required by the analysis pipeline. The sustained-vowel recordings were used exclusively for this matching step and were not used as model inputs. For VDR, this criterion resulted in the retention of 634 of 869 nonpathological speakers and 1354 of 1356 pathological speakers. For VDC, the analysis was restricted to the 4 most prevalent classes: nonpathological (n=634), hyperfunctional dysphonia (n=212), laryngitis (n=140), and recurrent laryngeal nerve paresis (n=213). In addition to providing sufficient sample sizes for supervised learning, these categories represent clinically relevant diagnostic scenarios frequently encountered in routine voice assessment and encompass functional, inflammatory, and neurological causes of dysphonia. All recordings were digitized at a sampling rate of 50 kHz with 16-bit resolution. Sentence recordings had durations ranging from 0.99 to 6.37 seconds (mean 2.17, SD 0.60 s).

**Figure 1.**
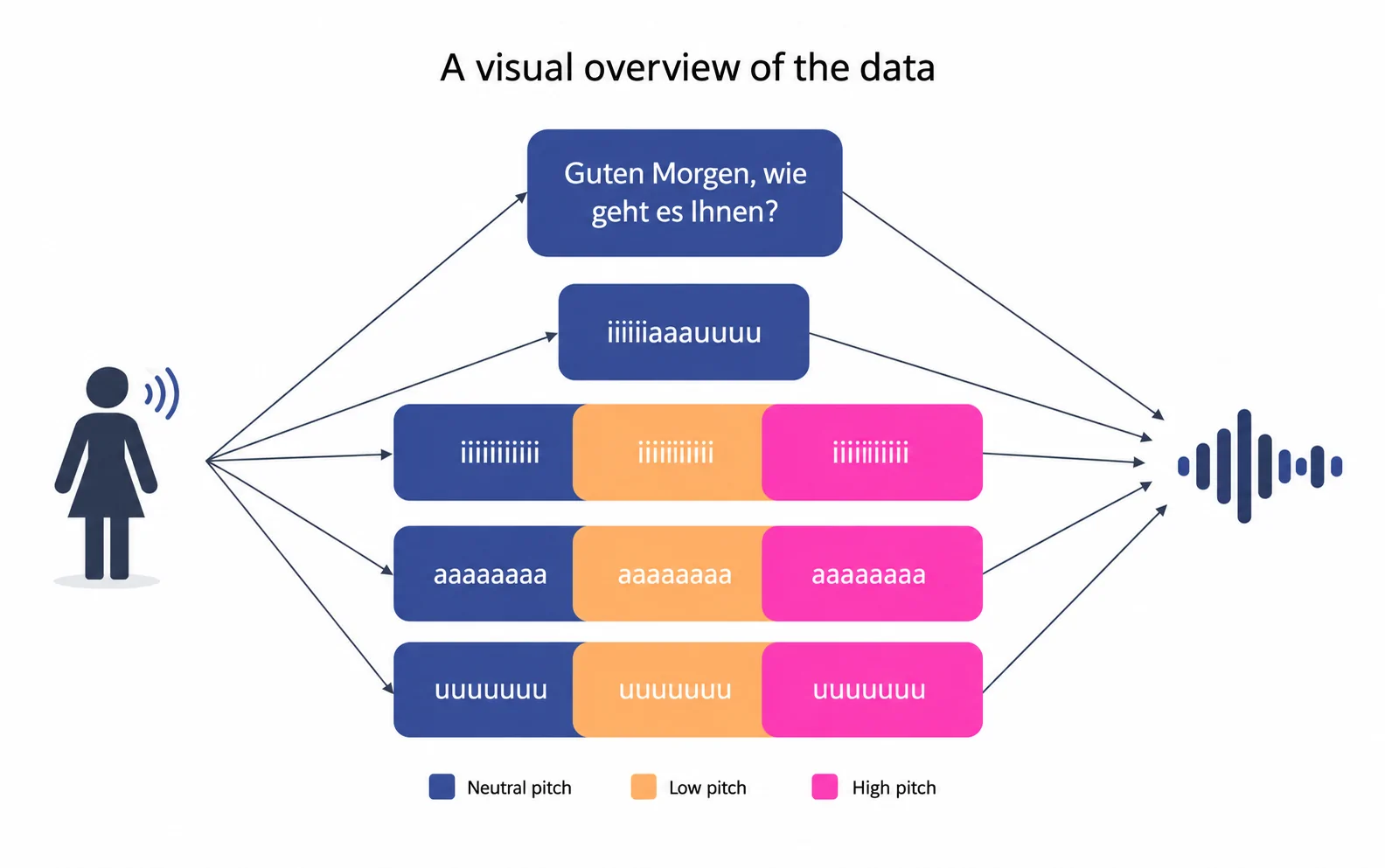
Dataset composition of the Saarbrücken Voice Database (SVD). The recording protocol comprised a standardized sentence (“Guten Morgen, wie geht es Ihnen”; English: “Good morning, how are you?”) and sustained vowel phonations (“i,” “a,” “u”) at neutral, low, and high pitch. The present analyses were restricted to standardized sentence recordings.

### Voice Signal Preprocessing and Augmentation

Data augmentation was applied exclusively to the training set, including synthetic recordings when used in a given experiment. Augmentation was performed prior to preprocessing because these operations directly affect signal length and amplitude. Augmentation was applied to 30% of the training samples using 6 strategies, with 1 strategy randomly selected for each augmented sample. The applied strategies included additive Gaussian noise (𝒩[0, 0.005]), time stretching by a factor of 1.2 (speed-up) or 0.8 (speed-down); pitch shifting by +4 semitones; and combinations of additive noise, pitch shifting, and either speed-up or speed-down. Following augmentation, all audio signals underwent a standardized preprocessing pipeline comprising padding or truncation, amplitude normalization to zero mean and unit variance, and resampling to 16 kHz. Based on the empirical length distribution of the sentence recordings, a fixed duration of 3 seconds was selected. Transformer-based models were trained directly on the resulting raw audio waveforms. In contrast, the remaining models, such as the long short-term memory (LSTM) model [17], were trained on mel-frequency cepstral coefficients (MFCCs) [18], extracted as the final preprocessing step.

### Synthetic Speech Generation and Evaluation

To augment the training data, synthetic speech samples were generated using TTS models. Two complementary TTS systems were evaluated: XTTS [16], a multilingual model, and Kartoffelbox [19], a German-only model. After the speaker-level train-test split, synthetic speech samples were generated exclusively for the training partition. For VDR, 1 synthetic counterpart was generated for each of the 1599 original training recordings per TTS model; for VDC, 1 synthetic counterpart was generated for each of the 959 original training recordings per TTS model. No synthetic samples were generated from the held-out test recordings. Across all experiments, the test set consisted exclusively of original recordings and remained unchanged. Based on these synthetic samples, 4 augmented training datasets were constructed (Table 1).

Across all experiments, the test set consisted exclusively of original recordings and remained identical to ensure fair and comparable evaluation. To assess the perceptual quality and diagnostic plausibility of the synthetic speech, a blinded listening evaluation was conducted. A total of 40 anonymized audio samples were selected such that neither the origin nor the diagnostic label could be inferred. The evaluation set included 10 nonpathological samples and 30 pathological samples, comprising hyperfunctional dysphonia, laryngitis, and recurrent laryngeal nerve paresis, with 10 samples per pathological category. For each category, 5 samples were original, and 5 were synthetic; all synthetic samples were generated directly from their corresponding original recordings.

The listening evaluation was performed by 1 board-certified otorhinolaryngologist and phoniatrician with more than 20 years of clinical experience assessing voice disorders. Each sample was independently evaluated for whether it was synthetic or original, whether it represented a pathological or nonpathological voice, and, for pathological samples, which diagnostic category best matched the perceived voice characteristics. The evaluation was conducted separately for synthetic samples generated by XTTS and Kartoffelbox. One synthetic sample of recurrent paresis was identified as defective and was excluded from both evaluations.

**Table 1.** Overview of the 4 datasets enriched with synthetic speech used in this study. Synthetic samples were generated only for the training partition, and all test sets contained only original recordings.

Dataset name	Dataset description
XTTS-np	It contains the original training data plus all XTTS-generated pathological and nonpathological training samples.
XTTS-n	It contains the original training data plus XTTS-generated nonpathological training samples only.
Kartoffelbox-np	It contains the original training data plus all Kartoffelbox-generated pathological and nonpathological training samples.
Kartoffelbox-n	It contains the original training data plus Kartoffelbox-generated nonpathological training samples only.

### VDR and VDC

To establish baseline performance for VDR, we evaluated a range of widely used machine learning and deep learning models that are well established in the voice analysis literature [7,9,20-22]. These included a support vector machine (SVM) [23], a ResNet-18 [24] implemented as a 2-dimensional convolutional neural network with batch normalization [25] and rectified linear unit (ReLU) activations [26], a recurrent neural network with 2 LSTM layers [17,27], and 3 transformer-based models: Hidden-Unit BERT (HuBERT) [28], Wav2Vec 2.0 [29], and WavLM [30]. The SVM, convolutional neural network, and LSTM models were trained using MFCCs [18], whereas the transformer-based models were trained directly on preprocessed raw audio waveforms. These architectures were selected to represent both classical feature-based approaches and modern self-supervised speech models. To assess the effect of synthetic speech augmentation, the LSTM and HuBERT models were additionally trained on data augmented with XTTS and Kartoffelbox. To combine the complementary strengths of different model types, we implemented an ensemble approach that integrates the outputs of 3 models. First, a conventional ensemble was constructed by averaging the individual models’ posterior probabilities. Building on this, we introduced a learnable fusion strategy, referred to as learnable fusion (LF), which assigns trainable weights to each model output, allowing the ensemble to adaptively emphasize the most informative representations (Figure 2).

The LF approach operates on the latent representations extracted from each model while keeping the individual model parameters fixed. Since these representations differ in dimensionality and temporal resolution, they were first aligned to a common representation space. The LSTM outputs were projected to match the dimensionality of the transformer representations, and all sequences were resampled to a fixed length. The fusion weights were initialized uniformly with equal weights and optimized during training. In an extended variant, additional information about the speaker’s sex was incorporated via a dedicated embedding. Speaker sex was encoded as a binary variable and mapped to a learnable embedding matrix of size 2 × 768. This embedding was broadcast across the temporal dimension and added element-wise to the fused representation before temporal mean pooling, rather than being concatenated (see Supplement S1 in Multimedia Appendix 1). The fused representation was subsequently passed to a classification head identical to the one used in the LSTM model. All ensemble approaches in VDR were applied to the LSTM and HuBERT models, both trained on the Kartoffelbox-np dataset, and to the HuBERT model trained on the Kartoffelbox-n dataset. Additional implementation details are provided in Section S1 in Multimedia Appendix 1. In addition, VDC experiments followed a methodology similar to that of the VDR task but focused on distinguishing among specific diagnostic categories. Baseline experiments were conducted using the LSTM, HuBERT, Wav2Vec 2.0, and WavLM models. These models were also trained on the Kartoffelbox-np augmented dataset to improve robustness. The ensemble approach with learnable fusion was applied to the HuBERT baseline model and to LSTM and Wav2Vec 2.0 models trained on Kartoffelbox-np data. For VDC, the output layer was adapted to predict 4 diagnostic classes.

**Figure 2.**
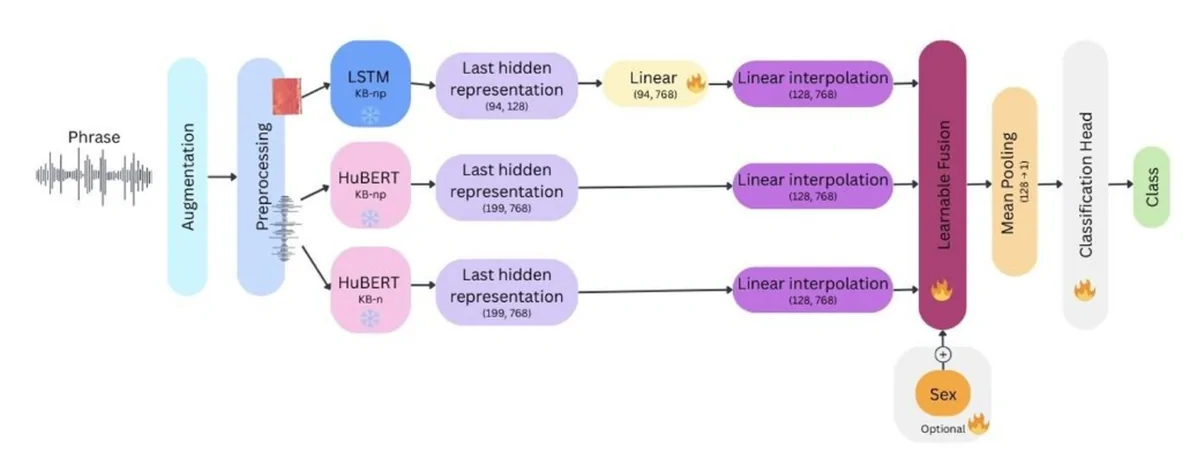
Overview of the learnable fusion (LF) approach for voice disorder recognition (VDR). Audio recordings are first augmented and preprocessed to provide both mel-frequency cepstral coefficient (MFCC) features for the long short-term memory (LSTM) model and raw waveforms for the transformer-based model. Latent representations from each model are extracted with frozen weights and aligned to a common dimension and sequence length. In the LF block, trainable weights determine each model’s contribution. Optionally, a speaker sex embedding can be added. The fused representation is mean-pooled and passed to a shared classification head. The architecture for VDC differs only in the pretrained models used (Hidden-Unit BERT [HuBERT] as the baseline, and LSTM and Wav2Vec 2.0 trained on Kartoffelbox-np).

### Training Procedure

For both VDR and VDC, a stratified 80:20 train-test split was performed to preserve class distributions. Splits were generated independently for each task. To prevent data leakage, all train-test splits were performed at the speaker level, ensuring that no recordings originating from the same individual were present in both the training and test sets. Consequently, model performance was evaluated exclusively on previously unseen speakers. All data augmentation procedures described above were applied exclusively to the training data, and absolute sample counts for the resulting datasets are summarized in Table 2.

Regarding the hyperparameters, we followed the methodology of Koudounas et al [7]. All models were trained for 100 epochs using a batch size of 8 and an initial learning rate of 1 × 10⁻⁴. A linear learning rate scheduler with 250 warm-up steps was used to stabilize early training. Optimization was performed using the Adam (adaptive moment estimation optimizer) [31] with cross-entropy loss [32]. To address class imbalance, class-weighted loss functions were used. For the LF and learnable fusion with sex embedding (LFS) approaches, the parameters of the individual base models were kept frozen during training. Only the parameters associated with the linear projection layers [33], fusion weights, optional sex embedding, and the classification head were updated.

**Table 2.** Absolute numbers of samples in train and test datasets used in voice disorder recognition (VDR) and voice disorder classification (VDC) tasks.

Dataset	Train samples, n	Test samples, n	Overall, n
VDR	1599	389	1988
VDC	959	240	1199

### Ablation and Component Comparisons

The experimental design included several comparisons of components. First, models trained on original data were compared with models trained on synthetic speech-augmented data to assess the contribution of TTS augmentation. Second, XTTS and Kartoffelbox augmentation were compared to evaluate the effect of TTS system choice. Third, augmentation with all synthetic samples (np) was compared with augmentation using only nonpathological synthetic samples (n). Fourth, individual model performance was compared with that of a simple, equally weighted ensemble to assess whether model combination alone improved performance. Fifth, LF was compared with the equal-weight ensemble to evaluate the contribution of learnable model weighting. Finally, LFS was compared with LF to isolate the contribution of the sex embedding.

### Statistical Analysis

Variables following a normal distribution are reported as mean SD, while nonnormally distributed variables are summarized as median with IQR. Normality was assessed using the Shapiro-Wilk test [34]. Model performance was evaluated using established classification metrics, including accuracy, *F*_1_-score, precision, recall, sensitivity, and specificity [7,20,35-37]. For binary classification, sensitivity corresponds to the recall of the positive class (pathological), whereas specificity corresponds to the recall of the negative class (nonpathological). In addition, the area under the receiver operating characteristic curve (AUC) [38] was reported for VDR to facilitate comparison with prior work [7]. Model performance was evaluated on the held-out test set. To quantify uncertainty, we applied nonparametric bootstrap resampling [39] of the test set with 100 iterations and reported performance as the bootstrap median with 95% bootstrap percentile CIs. Bootstrap resampling was used only for uncertainty estimation and not as independent observations for significance testing. For model comparisons, we calculated paired bootstrap distributions of metric differences using identical resampled test indices for both models. Differences are reported descriptively with 95% bootstrap percentile CIs. Intervals that cross zero were interpreted as not providing evidence of a clear performance difference. No hypothesis tests were applied to bootstrap resamples.

## Results

### Synthetic Speech Samples

The blinded perceptual evaluation revealed substantial differences in the perceptual quality of synthetic speech generated by the 2 TTS models. XTTS-generated synthetic samples were correctly identified as synthetic in 89.5% (17/19) of the cases, whereas only 52.6% (10/19) of the Kartoffelbox-generated synthetic samples were identified as synthetic, indicating that Kartoffelbox-generated speech was more difficult to distinguish from natural recordings. Recognition rates for original samples were comparable across both test sets (14/20, 70.0% for XTTS and 13/20, 65.0% for Kartoffelbox). An overview of the perceptual evaluation results is shown in Figure 3, with detailed results provided in Multimedia Appendix 1.

In the pooled XTTS evaluation, pathological-status identification was 65.5% (19/29) for pathological samples and 60.0% (6/10) for nonpathological samples, while pooled diagnostic-class assignment was 35.9% (14/39). In the pooled Kartoffelbox evaluation, pathological-status identification was 75.9% (22/29) for pathological samples and 80.0% (8/10) for nonpathological samples, while pooled diagnostic-class assignment was 51.3% (20/39). These findings suggest that Kartoffelbox-generated speech was perceptually more natural and diagnostically plausible in this blinded evaluation.

**Figure 3.**
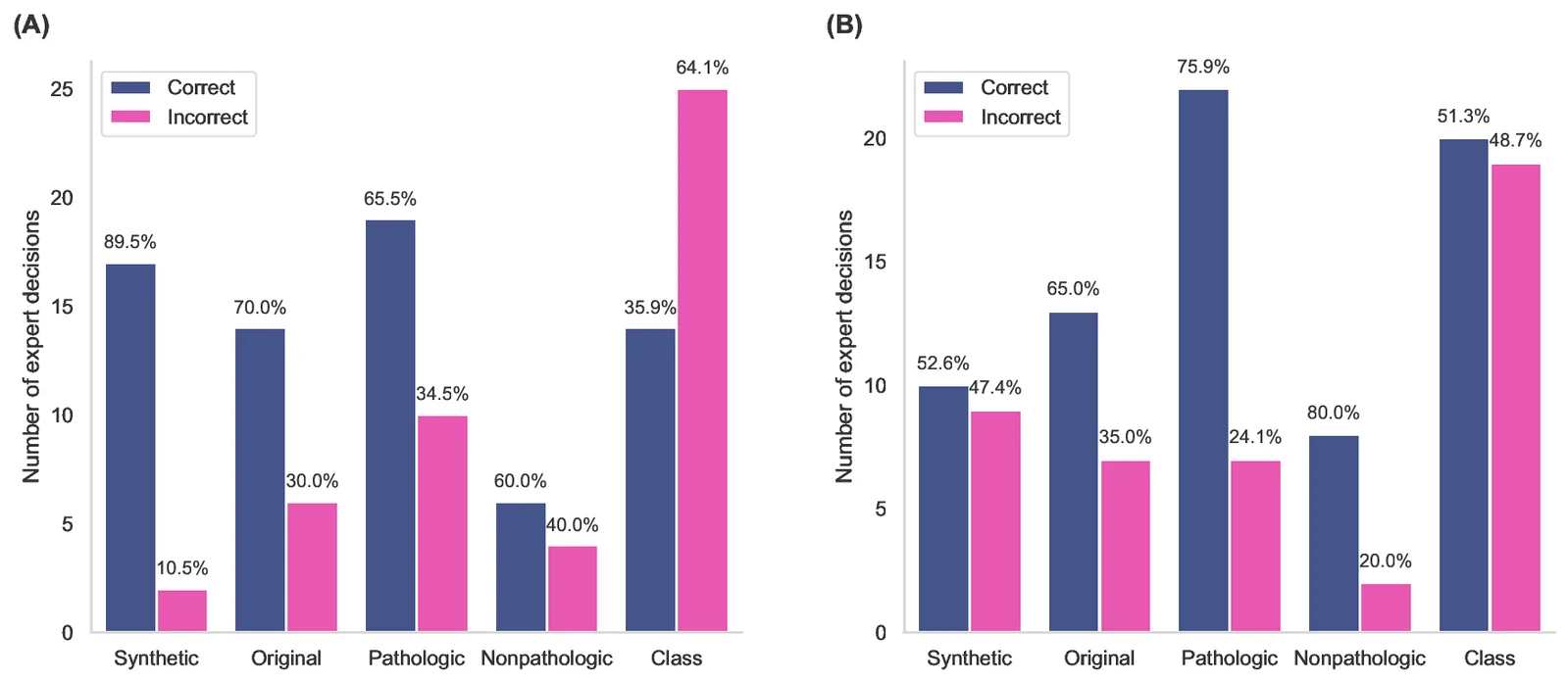
Results of the blinded perceptual evaluation of synthetic speech quality. The bar plots show the counts and proportions of correct and incorrect expert classifications across categories (synthetic, original, pathologic, nonpathologic, and overall class). Panel (A) shows results for XTTS-generated samples, and panel (B) shows results for Kartoffelbox-generated samples. Percentages above the bars indicate the relative proportion of correct and incorrect decisions within each category.

### VDR

Among baseline models trained on the original dataset, clear performance differences emerged. The SVM achieved the highest sensitivity but exhibited markedly lower specificity, indicating a tendency toward overdetection of pathological voices. In contrast, ResNet-18 and the LSTM model showed improved specificity at the cost of reduced sensitivity. Transformer-based models consistently outperformed classical deep learning and machine learning approaches. HuBERT achieved the most balanced performance, yielding the highest accuracy and *F*_1_-score, with closely matched sensitivity and specificity (Table 3).

Training with synthetic data further improved performance, particularly when augmentation was performed using Kartoffelbox-generated samples (Table 4). Both LSTM and HuBERT benefited from Kartoffelbox-based augmentation, with increases observed in sensitivity, accuracy, and *F*_1_-score. The HuBERT model trained on the Kartoffelbox-np dataset achieved the highest overall performance among single models, with a sensitivity of 0.88, an accuracy of 0.87, and an *F*_1_-score of 0.85. The effect of XTTS augmentation differed by architecture. For LSTM, XTTS-np showed a small shift toward lower sensitivity and higher specificity relative to the original training data, but the corresponding difference intervals included zero, indicating no clear change. In contrast, for HuBERT, XTTS-np increased sensitivity (0.848-0.886) while decreasing specificity (0.861-0.784), with both difference intervals excluding zero, indicating a shift toward detecting more pathological samples at the cost of more false positives.

**Table 3.** Baseline performance of the evaluated models for voice disorder recognition (VDR)^a^.

Model	Sensitivity (95% CI)	Specificity (95% CI)	Accuracy (95% CI)	*F*_1_-score (95% CI)
SVM^b^	0.915 (0.885‐0.950)	0.492 (0.394‐0.562)	0.776 (0.747‐0.814)	0.719 (0.669‐0.764)
ResNet-18	0.740 (0.694‐0.801)	0.769 (0.691‐0.822)	0.749 (0.705‐0.799)	0.732 (0.685‐0.782)
LSTM^c^	0.807 (0.775‐0.854)	0.815 (0.752‐0.875)	0.812 (0.774‐0.842)	0.797 (0.756‐0.827)
HuBERT^d^	0.848 (0.796‐0.886)	0.861 (0.799‐0.917)	0.849 (0.811‐0.887)	0.835 (0.795‐0.872)
Wav2Vec 2.0	0.882 (0.840‐0.921)	0.788 (0.729‐0.849)	0.852 (0.815‐0.886)	0.831 (0.791‐0.869)
WavLM	0.929 (0.899‐0.962)	0.656 (0.567‐0.756)	0.842 (0.795‐0.881)	0.806 (0.756‐0.856)

a Performance is reported as the bootstrap median for sensitivity, specificity, accuracy, and *F*_1_-score, based on 100 bootstrap resamples of the held-out test set.

b SVM: support vector machine.

c LSTM: long short-term memory.

d HuBERT: Hidden-Unit BERT (self-supervised speech model).

**Table 4.** Results for Hidden-Unit BERT (HuBERT) and long short-term memory (LSTM) trained on different datasets^a^.

Model	Data	Sensitivity (95% CI)	Specificity (95% CI)	Accuracy (95% CI)	*F*_1_-score (95% CI)
LSTM	Original	0.807 (0.775‐0.854)	0.815 (0.752‐0.875)	0.812 (0.774‐0.842)	0.797 (0.756‐0.827)
LSTM	XTTS-np	0.774 (0.725‐0.824)	0.851 (0.795‐0.912)	0.799 (0.762‐0.834)	0.785 (0.752‐0.822)
LSTM	XTTS-n	0.833 (0.782‐0.879)	0.765 (0.688‐0.835)	0.809 (0.781‐0.851)	0.789 (0.747‐0.832)
LSTM	Kartoffelbox-np	0.876 (0.844‐0.905)	0.818 (0.758‐0.878)	0.857 (0.828‐0.882)	0.838 (0.807‐0.869)
LSTM	Kartoffelbox-n	0.816 (0.769‐0.858)	0.773 (0.709‐0.835)	0.802 (0.760‐0.837)	0.780 (0.738‐0.818)
HuBERT	Original	0.848 (0.796‐0.886)	0.861 (0.799‐0.917)	0.849 (0.811‐0.887)	0.835 (0.795‐0.872)
HuBERT	XTTS-np	0.886 (0.835‐0.917)	0.784 (0.708‐0.847)	0.852 (0.810‐0.891)	0.832 (0.786‐0.875)
HuBERT	XTTS-n	0.879 (0.836‐0.919)	0.807 (0.740‐0.869)	0.857 (0.820‐0.892)	0.836 (0.801‐0.879)
HuBERT	Kartoffelbox-np	0.878 (0.838‐0.910)	0.846 (0.766‐0.907)	0.867 (0.829‐0.901)	0.850 (0.808‐0.887)
HuBERT	Kartoffelbox-n	0.908 (0.869‐0.935)	0.769 (0.703‐0.848)	0.862 (0.826‐0.898)	0.843 (0.798‐0.882)

a Performance is reported as the bootstrap median for sensitivity, specificity, accuracy, and *F*_1_-score, based on 100 bootstrap resamples of the held-out test set. Baseline results for the original dataset are included for comparison.

Using the LF approach (Table 5), the model learned weights of 0.35, 0.32, and 0.33 for HuBERT trained on Kartoffelbox-np, HuBERT trained on Kartoffelbox-n, and the LSTM model, respectively. This near-uniform weighting indicates that all 3 components contributed complementary information. Compared with individual baseline models, LF reduced the imbalance between sensitivity and specificity and achieved the highest accuracy among fusion approaches without sex embedding. Relative to HuBERT trained on the Kartoffelbox-np dataset, LF showed numerically higher accuracy and *F*_1_-score, while specificity was similar. The equal-weight ensemble achieved the highest AUC among the ensemble approaches. Paired bootstrap difference intervals are provided in Multimedia Appendix 1.

**Table 5.** Performance of ensemble-based approaches for the voice disorder recognition (VDR) task^a^.

Approach	Sensitivity (95% CI)	Specificity (95% CI)	Accuracy (95% CI)	*F*_1_-score (95% CI)	AUC^b^ (95% CI)
Ensemble	0.901 (0.859‐0.931)	0.820 (0.748‐0.884)	0.872 (0.835‐0.907)	0.855 (0.815‐0.893)	0.930 (0.900‐0.957)
LF^c^	0.891 (0.848‐0.923)	0.849 (0.775‐0.907)	0.874 (0.843‐0.907)	0.859 (0.821‐0.896)	0.912 (0.877‐0.941)
LFS^d^	0.877 (0.839‐0.914)	0.876 (0.806‐0.925)	0.877 (0.847‐0.908)	0.864 (0.832‐0.897)	0.922 (0.890‐0.955)

a Values are bootstrap medians with 95% bootstrap percentile CIs for sensitivity, specificity, accuracy, *F*_1_-score, and area under the receiver operating characteristic curve.

b AUC: area under the receiver operating characteristic curve.

c LF: learnable fusion.

d LFS: learnable fusion with sex embedding.

The LFS variant changed the balance between sensitivity and specificity. LFS achieved a sensitivity of 0.877 (95% CI 0.839‐0.914), a specificity of 0.876 (95% CI 0.806‐0.925), an accuracy of 0.877 (95% CI 0.847‐0.908), an *F*_1_-score of 0.864 (95% CI 0.832‐0.897), and an AUC of 0.922 (95% CI 0.890‐0.955). The learned fusion weights were similar to those of LF, with a slightly increased contribution from HuBERT trained on Kartoffelbox-np, indicating that incorporation of the sex embedding was associated with only modest changes in the relative contributions of the base-model representations (Figure 4).

Receiver operating characteristic analysis (Figure 4) showed that the equal-weight ensemble achieved the highest point-estimate AUC among the ensemble approaches. HuBERT slightly outperformed LFS at very low false-positive rates. Compared with LF, LFS showed higher specificity and AUC but lower sensitivity, while accuracy and *F*_1_-score were similar. Thus, incorporating sex information primarily shifted the balance between sensitivity and specificity rather than producing a clear overall performance gain.

**Figure 4.**
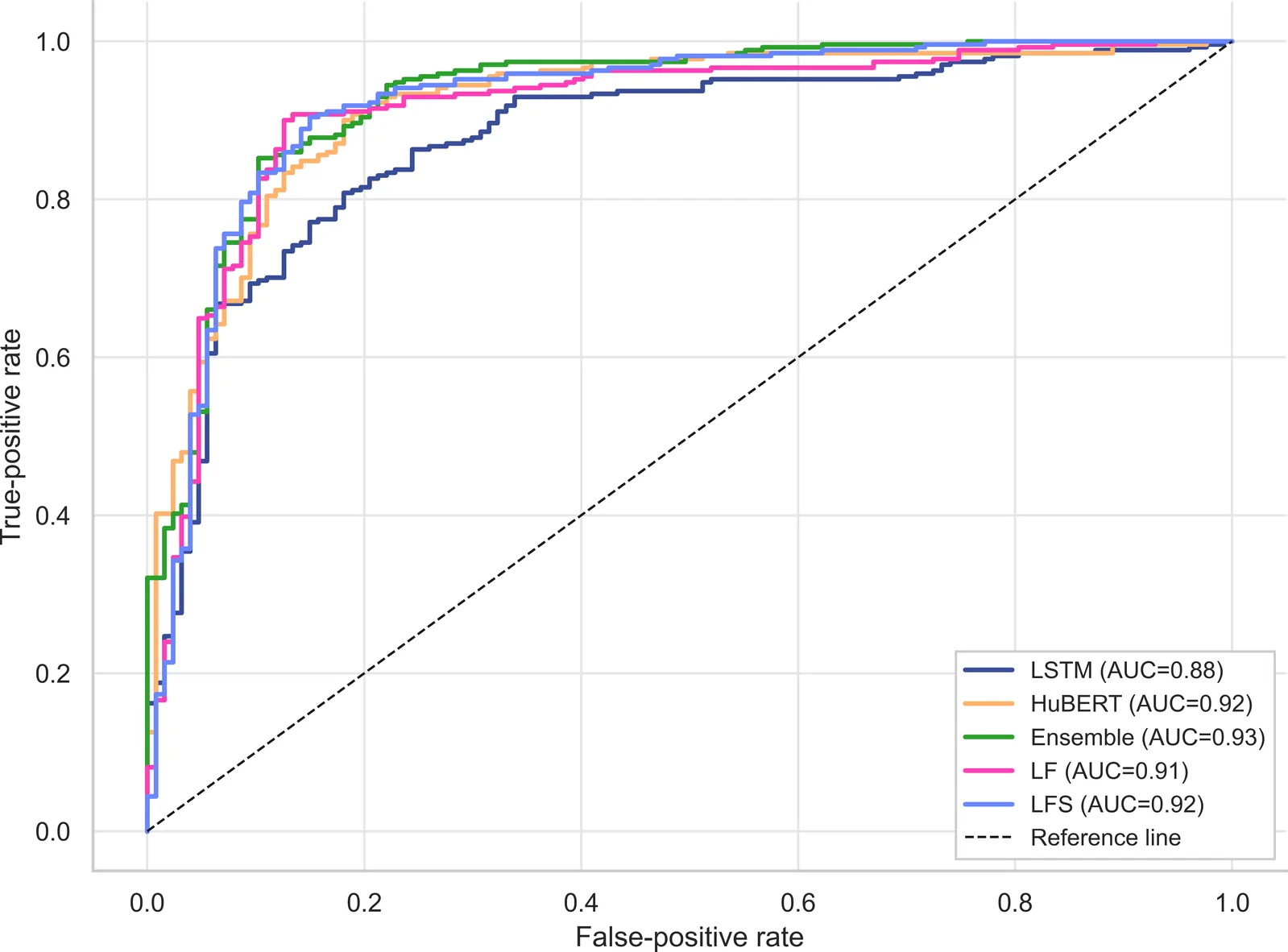
Receiver operating characteristic (ROC) curves illustrating the trade-off between true-positive and false-positive rates for the baseline models Hidden-Unit BERT (HuBERT) and long short-term memory (LSTM), the equal-weight ensemble, and the proposed learnable fusion approaches learnable fusion (LF) and learnable fusion with sex embedding (LFS). Reported area under the receiver operating characteristic curve (AUC) values indicate point estimates for the displayed ROC curves.

### VDC

For VDC, HuBERT again achieved the strongest baseline performance (median *F*_1_-score=0.63, accuracy=0.72), whereas WavLM showed the weakest results (median *F*_1_-score=0.37, accuracy=0.53). Across all models, the nonpathological class was classified with the highest reliability. Among pathological classes, recurrent paresis was consistently identified with the highest accuracy, while hyperfunctional dysphonia and laryngitis proved more challenging. Confusion matrix analysis (Figure 5) revealed systematic error patterns.

Hyperfunctional dysphonia was frequently misclassified as nonpathological. From a clinical perspective, this finding is plausible, as patients with hyperfunctional dysphonia may exhibit intermittent periods of near-normal voice production depending on vocal effort and speaking context. Such variability may reduce the acoustic distinction between hyperfunctional dysphonia and nonpathological speech, thereby contributing to the observed misclassification pattern. WavLM showed a tendency to overpredict recurrent paresis. For LSTM, both XTTS-based and Kartoffelbox-based augmentation improved overall *F*_1_-score and accuracy relative to the corrected original-data baseline, with the strongest improvement observed for Kartoffelbox. Kartoffelbox-based augmentation also improved overall performance for Wav2Vec 2.0 and WavLM, whereas augmentation did not improve overall performance for HuBERT relative to its original-data baseline. A notable exception was observed for the laryngitis class, where WavLM trained with Kartoffelbox augmentation failed to correctly identify any laryngitis samples in the class-specific analysis.

**Figure 5.**
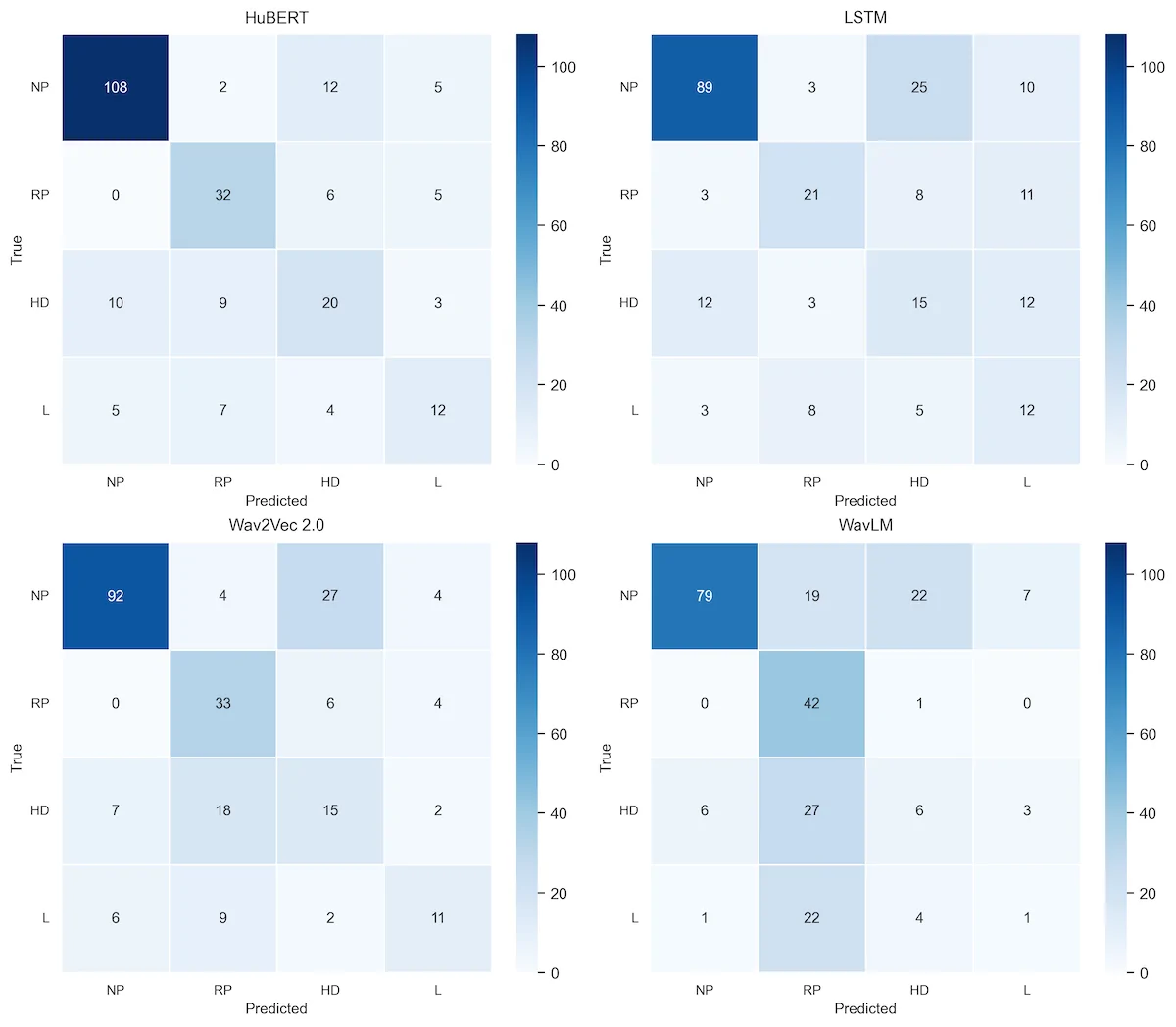
Confusion matrices for the 4 baseline models based on the full held-out test set. The nonpathological class shows the highest classification reliability, while recurrent paresis is the most consistently identified pathological category. HD: hyperfunctional dysphonia; HuBERT: Hidden-Unit BERT; L: laryngitis; LSTM: long short-term memory; NP: nonpathological; RP: recurrent paresis.

To contextualize the learnable fusion approach, we compared it with a simple equally weighted ensemble. For VDC, the equal-weight ensemble achieved an overall *F*_1_-score of 0.614 and an accuracy of 0.725. LF improved the overall *F*_1_-score to 0.659 and accuracy to 0.733, whereas LFS achieved an overall *F*_1_-score of 0.640 and accuracy of 0.721. The highest hyperfunctional dysphonia recall among LF and LFS was observed for LF (0.674), whereas LFS achieved a lower recall for this class (0.582). For laryngitis, LF improved *F*_1_-score compared with the equally weighted ensemble, but performance remained limited and was comparable to the best single-model augmented baselines. Within the LF model, laryngitis had the lowest *F*_1_-score among the 4 VDC classes.

## Discussion

### Principal Findings

In this study, we investigated deep learning–based approaches for VDR and VDC using the SVD. We evaluated a broad range of baseline and advanced models and systematically examined the impact of synthetic speech augmentation generated with 2 TTS systems, XTTS and Kartoffelbox. To ensure the clinical plausibility of the generated data, blinded perceptual evaluations were conducted, demonstrating that synthetic samples generated with Kartoffelbox more closely resembled natural speech than those produced with XTTS. Correspondingly, Kartoffelbox-based augmentation yielded more consistent improvements in both recognition and classification performance, underscoring the importance of synthetic data quality in medical speech applications. This alignment suggests that perceptually more natural and diagnostically plausible synthetic speech provides more effective augmentation for downstream model training. The contribution of this work is therefore not the isolated use of TTS, transformer models, or ensemble learning but the systematic comparison of their interaction under a shared experimental setting. The results show that augmentation effects were architecture- and class-dependent, supporting the need for integrated evaluation rather than assuming that synthetic speech uniformly improves robustness. This class-specific instability was most evident for WavLM trained with Kartoffelbox augmentation, which showed a complete performance collapse for laryngitis despite otherwise benefiting from augmentation, underscoring that synthetic data effects can be diagnosis-specific and model-specific.

Baseline experiments conducted on the original SVD confirmed the reproducibility of previously reported results. Classical machine learning approaches, such as SVMs, achieved high sensitivity but limited specificity, indicating a tendency to overidentify pathological voices. Deep learning models, particularly the LSTM and ResNet-18, provided a more balanced trade-off between sensitivity and specificity. Among the architectures evaluated, transformer-based models demonstrated superior performance, with HuBERT consistently outperforming Wav2Vec 2.0 and WavLM across both VDR and VDC tasks. This finding supports prior evidence that self-supervised speech representations capture diagnostically relevant acoustic features more effectively than conventional feature-based approaches [9,40,41].

Combining heterogeneous models through ensemble learning provided a balanced overall performance. The proposed learnable fusion strategies enabled adaptive weighting of model contributions. LF showed small numerical improvements over equal weight averaging for several threshold-dependent performance metrics, whereas LFS did not consistently exceed the equal weight ensemble. The equal weight ensemble achieved the highest AUC among the ensemble approaches. These differences should therefore be interpreted cautiously because CIs overlapped across the fusion approaches. The incorporation of speaker sex as an additional embedding changed the sensitivity-specificity balance in VDR but did not consistently improve VDC performance relative to LF. This finding aligns with established knowledge that biological sex influences vocal characteristics and the physiology of voice production and highlights the potential relevance of clinically relevant metadata for AI-based voice analysis pipelines [42,43]. The results of the blinded perceptual evaluation corroborated these findings, supporting the reliability of the proposed models and demonstrating their potential to provide objective assessments less susceptible to human bias.

The observed changes associated with incorporating speaker sex information also highlight the potential value of investigating routinely available clinical metadata in AI-based voice assessment workflows. In practical deployment scenarios, such information could be obtained automatically from structured electronic health records or Fast Healthcare Interoperability Resources–based clinical information systems or alternatively entered manually during voice acquisition. Consequently, the incorporation of such metadata is unlikely to impose substantial additional workflow burden and warrants further investigation as a potential source of complementary information for AI-based voice assessment. Compared with prior SVD-based studies, our baseline models achieved performance largely comparable to that of the prior studies. Koudounas et al [7] reported an accuracy of 0.80 for a 2-dimensional convolutional neural network and 0.86 for HuBERT. In our experiments, ResNet-18 achieved an accuracy of 0.76, while HuBERT reached 0.85. The slightly lower performance of the convolutional model may be attributable to architectural or input-representation differences that were not fully specified in prior work. Their ensemble approach achieved an accuracy of 0.91, representing the highest reported performance for VDR on the SVD. Our LFS approached this benchmark in accuracy and added interpretability through adaptive weighting.

Performance differences between the models evaluated on the SVD and those reported on the VOICED dataset were consistently observed, with higher performance on the VOICED dataset. This discrepancy is likely explained by fundamental differences in dataset composition. VOICED contains only a small number of pathological categories, resulting in relatively homogeneous pathological samples. In contrast, the SVD includes 71 pathological diagnostic categories plus a nonpathological category, introducing substantial acoustic and pathophysiological variability. While this heterogeneity increases task complexity and limits achievable performance, it more accurately reflects real-world clinical populations and therefore represents a more realistic benchmark for clinical applicability. The binary VDR task should therefore be interpreted as a screening-oriented task. The pathological class contains substantial acoustic and pathophysiological heterogeneity, and the model output cannot identify the underlying diagnosis. Its potential use lies in flagging recordings for further specialist assessment, not in replacing laryngological diagnosis. This suggests that the approach may be suited for further evaluation as an initial screening or preassessment tool in clinical or telemedicine settings, consistent with the broader potential of deep learning–based decision support in health care [44]. Such systems could support earlier identification of potential voice disorders and facilitate timely referral to specialized care. In screening scenarios, operating points may be selected to prioritize sensitivity and minimize missed pathological cases, while accepting a corresponding increase in false-positive results [45]. However, thresholds should be calibrated to the intended clinical workflow to balance referral burden against the risk of delayed diagnosis. Importantly, the proposed approach is not intended to replace professional medical diagnosis but rather to complement clinical workflows by providing scalable, objective decision support.

While objective acoustic analysis provides valuable diagnostic information, it may not fully capture the patient’s perceived vocal impairment. Previous studies have demonstrated that subjective voice complaints, voice-related handicap, and participation restrictions may differ substantially across etiological groups despite similar acoustic manifestations. In particular, Lee et al [46] reported differences in voice activity and participation profiles across etiological categories of voice disorders, highlighting the multidimensional nature of dysphonia. Because subjective voice complaints and functional voice-related handicap may differ substantially depending on the etiology of dysphonia, future studies should combine objective acoustic classification approaches with subjective and patient-reported voice outcome measures to better reflect the multidimensional nature of voice disorders [46]. Such multimodal assessment strategies may facilitate more comprehensive clinical decision-making and improve the clinical relevance of AI-assisted voice analysis [47].

It should be noted that the used TTS models do not explicitly simulate underlying pathophysiological mechanisms such as inflammation, vocal fold paralysis, or hyperfunctional phonation. Instead, they learn statistical acoustic patterns present in the training data, including characteristics associated with pathological speech. Consequently, the generated samples should be interpreted as distributional augmentations rather than physiologically grounded simulations of disease processes. Furthermore, although synthetic speech augmentation is frequently discussed in the context of class imbalance, the primary objective of the present work was not to balance class distributions but to investigate whether synthetic speech could serve as an additional source of training data to improve model robustness and generalization.

### Limitations and Future Work

Several limitations should be considered when interpreting these results. First, the SVD was collected between the late 1990s and early 2000s. Advances in recording technology since then may allow for higher-fidelity capture of acoustic features, and it remains unclear how models trained on legacy data will generalize to contemporary recordings. Second, although the SVD encompasses a wide range of pathological conditions, many diagnostic categories are represented by only a few samples, leading to pronounced class imbalance. Even with stratified splitting and class-weighted loss functions, models cannot learn robust decision boundaries for severely underrepresented classes, rendering performance estimates for these categories statistically fragile. Third, the standardized sentence used in the SVD is relatively short. In clinical practice, voice assessment often relies on longer utterances or sustained phonation, as certain pathological features emerge only over time. The use of a fixed, short sentence may therefore limit the model’s ability to capture clinically relevant characteristics. In addition, the lower retention of nonpathological than pathological speakers in the VDR cohort may have introduced selection bias and should be considered when interpreting the VDR performance estimates. Finally, although the overall dataset is balanced by biological sex, the subset of classes used for VDC is skewed toward female speakers, potentially biasing classification performance.

Future work should focus on collecting additional data from longer speech samples, improving class balance, and using contemporary recording conditions to further enhance the model’s robustness and generalizability. In addition, a greater number of synthetic speech samples should be verified by multiple clinical experts to provide a more diverse assessment of the TTS capabilities with respect to pathological voice signals. The perceptual evaluation of synthetic speech quality was performed by a single board-certified otorhinolaryngologist and phoniatrician with more than 20 years of clinical experience in voice-disorder assessment. Although this provided expert clinical assessment, the use of a single rater introduces potential subjective bias and precludes assessment of interrater agreement using established metrics such as Cohen κ or Fleiss’ κ. Future studies should include multiple independent experts and larger listening cohorts to improve robustness and enable formal reliability analyses. Furthermore, future work should investigate the use of modern TTS systems to generate longer and more natural connected speech samples. Such recordings may better capture temporal, prosodic, and contextual characteristics of pathological voices than short, standardized utterances and could therefore provide additional information for both model training and evaluation.

Finally, although the proposed LF framework demonstrated strong performance and interpretability, alternative multimodel integration approaches, including attention-based fusion, stacking, and meta-learning strategies, may offer additional benefits [48,49]. Systematic comparisons between these approaches and the proposed framework represent an important direction for future research.

### Conclusions

Overall, the results of this study show that automated detection of pathological voices can be achieved with high performance using the evaluated models, whereas classification into specific diagnostic categories remains more challenging. The applied methods demonstrate the potential of AI-based approaches for recognizing voice disorders from speech recordings. However, these approaches are not intended to replace established clinical diagnostic procedures, such as laryngostroboscopy, but may serve as complementary tools within clinical workflows.

## Supplementary material

10.2196/94285 Multimedia Appendix 1 Supplementary technical details and extended results, including learnable fusion implementation, synthetic speech quality evaluation, supplementary figures, bootstrap performance tables, paired comparison intervals, and the diagnostic-label distribution of the Saarbrücken Voice Database.

## Abbreviations

Adam: adaptive moment estimation optimizer
AUC: area under the receiver operating characteristic curve
HuBERT: Hidden-Unit BERT
LF: learnable fusion
LFS: learnable fusion with sex embedding
LSTM: long short-term memory
MFCC: mel-frequency cepstral coefficient
ReLU: rectified linear unit
SVD: Saarbrücken Voice Database
SVM: support vector machine
TTS: text-to-speech
VDC: voice disorder classification
VDR: voice disorder recognition
VOICED: Voice Icar Federico
