# Supplementary material for: Detection of Pathological Voice Samples Using a Text-to-Speech–Inspired Approach With Multiple Model Architectures: Method Development and Comparative Evaluation Study
Source: J Med Internet Res. 2026 Oct 19;28:e94285. doi: 10.2196/94285 (PMC13679908; doi:10.2196/94285)
Supplement: Supplementary file 1 [file jmir-v28-e94285-s001.docx]

# **Supplementary Material**

### **S1. Technical Details of the Learnable Fusion Approach**

This section provides additional technical details on the Learnable Fusion (LF) and Learnable Fusion with Sex Embedding (LFS) approaches, focusing on the implementation and mathematical formulation of the ensemble weighting mechanism. LF operates on latent representations extracted from multiple pretrained base models, with all base-model parameters frozen. Let *h1*, *h2*, and *h3* denote the processed last hidden representations of HuBERT trained on the Kartoffelbox-np dataset, HuBERT trained on the Kartoffelbox-n dataset, and the LSTM trained on the Kartoffelbox-np dataset, respectively. To ensure a probabilistic and interpretable weighting of model contributions, a softmax function is applied to the learnable weight vector *w*:

*w = softmax(w)*

The fused ensemble representation is then computed as a convex combination of the individual model representations:

*h_ensemble = w1 · h1 + w2 · h2 + w3 · h3*

This formulation ensures that all weights are non-negative and sum to one, allowing the model to adaptively emphasize the most informative representations during training. In the LFS variant, speaker sex is encoded as a binary categorical variable and mapped through a learnable embedding matrix Eₛₑₓ ∈ ℝ^(2 × 768). The resulting embedding vector is broadcast along the temporal dimension to match the fused latent sequence and is added to the fused representation before temporal mean pooling:

hₗᶠˢ = hₑₙₛₑₘᵦₗₑ + Eₛₑₓ(s)

where s denotes the binary speaker-sex label. Thus, the sex embedding is integrated additively rather than concatenated with the acoustic representation. The mean-pooled representation is subsequently passed to the shared classification head.

### For the LSTM baseline and its use in the fusion models, audio waveforms were converted into mel-frequency cepstral coefficient (MFCC) sequences with 20 coefficients, an FFT length (n_fft) of 512, and 40 mel filters. The remaining MFCC parameters followed the corresponding librosa defaults. In this implementation, both the hop length and window length were 512. The LSTM architecture used an input dimension of 20, a hidden dimension of 128, two recurrent layers, and a dropout rate of 0.2. Before fusion, the 128-dimensional LSTM representations were linearly projected to 768 dimensions to match the transformer representations. Sequence lengths were aligned to 128 time steps before fusion. The classical support vector machine (SVM) baseline used a radial basis function (RBF) kernel with C=1.0 and gamma="scale".

### **S2. Detailed Results of the Evaluation of Synthetic Speech Quality**

#### A detailed analysis of the blinded perceptual evaluation was conducted separately for synthetic and original speech samples to better understand differences in perceptual realism and diagnostic plausibility. Evaluation outcomes distinguish between correct identification of pathological status (pathological vs. non-pathological), correct assignment to the specific diagnostic class, and correct identification of sample origin (synthetic vs. original). Percentages are calculated for the corresponding synthetic, original, or pooled sample subset, as specified.

#### **S2.1 XTTS-Based Synthetic Speech**

For XTTS-generated synthetic samples, 71.4% of pathological recordings were correctly identified as pathological, while 40.0% of non-pathological recordings were correctly identified as non-pathological. Among the synthetic pathological samples, 26.3% were correctly assigned to their specific diagnostic class (Figure S2.1).

In the pooled XTTS evaluation, 89.5% of synthetic samples and 70.0% of original samples were correctly identified with respect to sample origin. Pathological-status identification was 65.5% for pathological samples and 60.0% for non-pathological samples, while pooled diagnostic-class assignment was 35.9%. Correct diagnostic-class assignment was 45.0% among the original XTTS evaluation samples (Figure S2.1).

Class-wise analysis revealed distinct patterns (Figure S2.3). For laryngitis, sample-origin identification was correct for 100% of synthetic and 80.0% of original samples. In this class, 70.0% were correctly identified as pathological, while 10.0% were correctly assigned to the specific diagnostic class. For hyperfunctional dysphonia, sample-origin identification was correct for 100% of synthetic and 40.0% of original samples. In this class, 30.0% were correctly identified as pathological, while 10.0% were correctly assigned to the specific diagnostic class. For recurrent paresis, sample-origin identification was correct for 75.0% of synthetic and 80.0% of original samples. All recurrent-paresis samples were correctly identified as pathological, and 66.7% were correctly assigned to the specific diagnostic class. Among non-pathological samples, sample-origin identification was correct for 80.0% of both synthetic and original recordings, while 60.0% were correctly identified as non-pathological.

#### **S2.2 Kartoffelbox-Based Synthetic Speech**

For Kartoffelbox-generated synthetic samples, 64.3% of pathological recordings were correctly identified as pathological, and 80.0% of non-pathological recordings were correctly identified as non-pathological. Correct diagnostic-class assignment among synthetic samples was 52.6%. For original samples in the Kartoffelbox evaluation, 86.7% of pathological recordings were correctly identified as pathological and 80.0% of non-pathological recordings as non-pathological, while 50.0% were correctly assigned to the specific diagnostic class (Figure S2.2).

In the pooled Kartoffelbox evaluation, 52.6% of synthetic samples and 65.0% of original samples were correctly identified with respect to sample origin. Overall pathological-status identification was 75.9% for pathological samples and 80.0% for non-pathological samples, and pooled diagnostic-class assignment was 51.3%.

Class-wise analysis revealed different patterns across diagnostic groups (Figure S2.4). For laryngitis, sample-origin identification was correct for 60.0% of synthetic and 60.0% of original samples. In this class, 90.0% were correctly identified as pathological, while 20.0% were correctly assigned to the specific diagnostic class. For hyperfunctional dysphonia, sample-origin identification was correct for 80.0% of synthetic and 60.0% of original samples. In this class, 50.0% were correctly identified as pathological, while 40.0% were correctly assigned to the specific diagnostic class. For recurrent paresis, sample-origin identification was correct for 25.0% of synthetic and 80.0% of original samples. In this class, 88.9% were correctly identified as pathological, and 55.6% were correctly assigned to the specific diagnostic class. Among non-pathological samples, sample-origin identification was correct for 40.0% of synthetic and 80.0% of original recordings, while 80.0% were correctly identified as non-pathological.

Across both XTTS and Kartoffelbox evaluations, hyperfunctional dysphonia was frequently misclassified as non-pathological, and laryngitis showed limited diagnostic-class assignment. Recurrent paresis showed the highest diagnostic-class assignment among the pathological categories in both evaluations (Figures S2.3 and S2.4).

**Figure S2.1**. Results of the blinded perceptual evaluation for XTTS-based synthetic speech, shown separately for original (a) and synthetic (b) samples. Results distinguish sample-origin identification, pathological-status identification, and diagnostic-class assignment.

**Figure S2.2**. Results of the blinded perceptual evaluation for Kartoffelbox-based synthetic speech, shown separately for synthetic and original samples. Results distinguish sample-origin identification, pathological-status identification, and diagnostic-class assignment, with performance differences varying across evaluation outcomes rather than consistently favoring original recordings.

**Figure S2.3**. Class-wise results of the XTTS-based perceptual evaluation. Results distinguish sample-origin identification, pathological-status identification, and diagnostic-class assignment. Recurrent paresis showed the highest pathological-status identification and diagnostic-class assignment among the pathological categories, whereas laryngitis and hyperfunctional dysphonia showed limited diagnostic-class assignment.

**Figure S2.4**. Class-wise results of the Kartoffelbox-based perceptual evaluation. Results distinguish sample-origin identification, pathological-status identification, and diagnostic-class assignment. Recurrent paresis and laryngitis were frequently identified as pathological, whereas hyperfunctional dysphonia showed lower pathological-status identification. Synthetic recurrent-paresis and non-pathological samples were frequently perceived as original recordings, and diagnostic-class assignment remained limited for laryngitis and hyperfunctional dysphonia.

## Supplementary Figures

**Figure S3.1**. Boxplots of overall performance metrics across baseline models. HuBERT achieved the highest overall scores, whereas WavLM showed the lowest overall performance.

**Figure S3.2. Class-wise baseline and augmentation performance for the Voice Disorder Classification (VDC) task. Panel A shows class-wise precision, recall, and F1-score across the four baseline models. Panel B shows class-wise and overall F1-score distributions for the baseline and synthetic-data augmentation variants. For LSTM, both XTTS-based and Kartoffelbox-based augmentation improved overall F1-score relative to the corrected original-data baseline, with the strongest improvement observed for Kartoffelbox. HuBERT performance decreased with both augmentation strategies, with a smaller decline observed for Kartoffelbox. Wav2Vec 2.0 and WavLM benefited from Kartoffelbox-based augmentation. Abbreviations: hyperfunctional dysphonia (HD), laryngitis (L), non-pathological (NP), recurrent paresis (RP).**

**Figure S3.3**. Class-wise performance comparison of the HuBERT baseline, equally weighted ensemble, and learnable fusion approaches. Learnable fusion (LF) showed numerical improvements for several performance metrics, although these differences were generally modest. **Abbreviations:** hyperfunctional dysphonia (HD), laryngitis (L), non-pathological (NP), recurrent paresis (RP).

| **Class** | **Precision** | **Recall** | **F1** | **Accuracy** |
| --- | --- | --- | --- | --- |
| NP | 0.841 (0.738–0.891) | 0.697 (0.622–0.777) | 0.763 (0.685–0.814) | — |
| RP | 0.609 (0.409–0.747) | 0.489 (0.356–0.659) | 0.539 (0.400–0.674) | — |
| HD | 0.283 (0.137–0.403) | 0.353 (0.205–0.507) | 0.315 (0.161–0.444) | — |
| L | 0.271 (0.149–0.410) | 0.431 (0.267–0.616) | 0.333 (0.207–0.469) | — |
| **Overall** | **0.497 (0.445–0.559)** | **0.492 (0.433–0.567)** | **0.483 (0.423–0.551)** | **0.573 (0.500–0.629)** |

**Table S4.1.** Performance of the LSTM baseline model for voice disorder classification using phrase recordings. Values are reported as bootstrap medians with 95% confidence intervals in parentheses, based on 100 bootstrap resamples of the held-out test set. Metrics are shown separately for each diagnostic class and for overall performance. **Abbreviations:** hyperfunctional dysphonia (HD), laryngitis (L), non-pathological (NP), recurrent paresis (RP).

| **Class** | **Precision** | **Recall** | **F1** | **Accuracy** |
| --- | --- | --- | --- | --- |
| NP | 0.878 (0.840–0.926) | 0.849 (0.794–0.914) | 0.864 (0.831–0.901) | — |
| RP | 0.637 (0.490–0.773) | 0.750 (0.595–0.845) | 0.687 (0.568–0.780) | — |
| HD | 0.488 (0.292–0.615) | 0.477 (0.308–0.641) | 0.478 (0.306–0.580) | — |
| L | 0.500 (0.315–0.735) | 0.428 (0.285–0.627) | 0.477 (0.308–0.608) | — |
| Overall | 0.624 (0.550–0.700) | 0.632 (0.560–0.697) | 0.625 (0.555–0.689) | 0.721 (0.675–0.769) |

**Table S4.2.** Performance of the HuBERT baseline model for voice disorder classification using phrase recordings. Values are reported as the bootstrap median with 95% confidence intervals in parentheses, based on 100 bootstrap resamples of the held-out test set. Metrics are shown separately for each diagnostic class and overall performance. **Abbreviations:** hyperfunctional dysphonia (HD), laryngitis (L), non-pathological (NP), recurrent paresis (RP).

| Class | Precision | Recall | F1 | Accuracy |
| --- | --- | --- | --- | --- |
| NP | 0.884 (0.831–0.930) | 0.721 (0.632–0.791) | 0.794 (0.733–0.838) | — |
| RP | 0.518 (0.389–0.660) | 0.785 (0.646–0.899) | 0.619 (0.488–0.721) | — |
| HD | 0.306 (0.188–0.424) | 0.350 (0.206–0.516) | 0.322 (0.190–0.446) | — |
| L | 0.558 (0.339–0.770) | 0.412 (0.245–0.603) | 0.479 (0.292–0.631) | — |
| Overall | 0.562 (0.490–0.646) | 0.570 (0.490–0.646) | 0.552 (0.464–0.628) | 0.637 (0.571–0.688) |

**Table S4.3.** Performance of the Wav2Vec 2.0 baseline model for voice disorder classification using phrase recordings. Values are reported as the bootstrap median with 95% confidence intervals in parentheses, based on 100 bootstrap resamples of the held-out test set. Metrics are shown separately for each diagnostic class and overall performance. **Abbreviations:** hyperfunctional dysphonia (HD), laryngitis (L), non-pathological (NP), recurrent paresis (RP).

| **Class** | **Precision** | **Recall** | **F1** | **Accuracy** |
| --- | --- | --- | --- | --- |
| NP | 0.916 (0.862–0.970) | 0.623 (0.556–0.698) | 0.741 (0.678–0.793) | — |
| RP | 0.378 (0.289–0.476) | 0.977 (0.933–1.000) | 0.543 (0.445–0.642) | — |
| HD | 0.188 (0.085–0.328) | 0.143 (0.059–0.247) | 0.164 (0.069–0.280) | — |
| L | 0.083 (0.000–0.311) | 0.034 (0.000–0.111) | 0.049 (0.000–0.147) | — |
| Overall | 0.393 (0.339–0.480) | 0.445 (0.412–0.485) | 0.373 (0.332–0.430) | 0.533 (0.475–0.592) |

**Table S4.4.** Performance of the WavLM baseline model for voice disorder classification using phrase recordings. Values are reported as the bootstrap median with 95% confidence intervals in parentheses, based on 100 bootstrap resamples of the held-out test set. Metrics are shown separately for each diagnostic class and overall performance. **Abbreviations:** hyperfunctional dysphonia (HD), laryngitis (L), non-pathological (NP), recurrent paresis (RP).

| **Class** | **Precision** | **Recall** | **F1** | **Accuracy** |
| --- | --- | --- | --- | --- |
| NP | 0.671 (0.601–0.722) | 0.929 (0.868–0.966) | 0.780 (0.720–0.820) | — |
| RP | 1.000 (0.774–1.000) | 0.128 (0.037–0.256) | 0.226 (0.071–0.408) | — |
| HD | 0.500 (0.301–0.656) | 0.325 (0.194–0.507) | 0.398 (0.237–0.559) | — |
| L | 0.257 (0.097–0.412) | 0.254 (0.115–0.427) | 0.252 (0.109–0.407) | — |
| Overall | 0.595 (0.522–0.673) | 0.409 (0.356–0.478) | 0.415 (0.341–0.498) | 0.608 (0.535–0.671) |

**Table S4.5.** Performance of the LSTM model for voice disorder classification using additional synthetic data generated with XTTS. Values are reported as the bootstrap median with 95% confidence intervals in parentheses, based on 100 bootstrap resamples of the held-out test set. Metrics are shown separately for each diagnostic class and overall performance. **Abbreviations:** hyperfunctional dysphonia (HD), laryngitis (L), non-pathological (NP), recurrent paresis (RP).

| **Class** | **Precision** | **Recall** | **F1** | **Accuracy** |
| --- | --- | --- | --- | --- |
| NP | 0.874 (0.800–0.926) | 0.731 (0.631–0.790) | 0.798 (0.724–0.832) | — |
| RP | 0.631 (0.498–0.760) | 0.560 (0.381–0.684) | 0.594 (0.453–0.690) | — |
| HD | 0.399 (0.296–0.515) | 0.632 (0.500–0.800) | 0.479 (0.368–0.591) | — |
| L | 0.299 (0.124–0.480) | 0.312 (0.105–0.529) | 0.311 (0.112–0.472) | — |
| Overall | 0.549 (0.501–0.612) | 0.556 (0.499–0.635) | 0.540 (0.491–0.611) | 0.637 (0.581–0.673) |

**Table S4.6.** Performance of the LSTM model for voice disorder classification using additional synthetic data generated with Kartoffelbox. Values are reported as the bootstrap median with 95% confidence intervals in parentheses, based on 100 bootstrap resamples of the held-out test set. Metrics are shown separately for each diagnostic class and overall performance. **Abbreviations:** hyperfunctional dysphonia (HD), laryngitis (L), non-pathological (NP), recurrent paresis (RP).

| **Class** | **Precision** | **Recall** | **F1** | **Accuracy** |
| --- | --- | --- | --- | --- |
| NP | 0.864 (0.813–0.919) | 0.837 (0.781–0.902) | 0.849 (0.804–0.895) | — |
| RP | 0.492 (0.374–0.630) | 0.804 (0.645–0.908) | 0.612 (0.492–0.721) | — |
| HD | 0.418 (0.242–0.583) | 0.269 (0.120–0.408) | 0.331 (0.167–0.438) | — |
| L | 0.509 (0.314–0.715) | 0.364 (0.214–0.555) | 0.417 (0.264–0.572) | — |
| Overall | 0.573 (0.479–0.655) | 0.574 (0.491–0.633) | 0.556 (0.471–0.621) | 0.677 (0.613–0.733) |

**Table S4.7.** Performance of the HuBERT model for voice disorder classification using additional synthetic data generated with XTTS. Values are reported as the bootstrap median with 95% confidence intervals in parentheses, based on 100 bootstrap resamples of the held-out test set. Metrics are shown separately for each diagnostic class and overall performance. **Abbreviations:** hyperfunctional dysphonia (HD), laryngitis (L), non-pathological (NP), recurrent paresis (RP).

| **Class** | **Precision** | **Recall** | **F1** | **Accuracy** |
| --- | --- | --- | --- | --- |
| NP | 0.855 (0.808–0.899) | 0.875 (0.828–0.927) | 0.867 (0.827–0.903) | — |
| RP | 0.543 (0.418–0.679) | 0.742 (0.599–0.834) | 0.628 (0.518–0.722) | — |
| HD | 0.453 (0.271–0.620) | 0.300 (0.164–0.462) | 0.355 (0.202–0.507) | — |
| L | 0.566 (0.388–0.725) | 0.459 (0.317–0.662) | 0.519 (0.367–0.660) | — |
| Overall | 0.593 (0.540–0.679) | 0.596 (0.543–0.665) | 0.587 (0.531–0.660) | 0.700 (0.656–0.756) |

**Table S4.8.** Performance of the HuBERT model for voice disorder classification using additional synthetic data generated with Kartoffelbox. Values are reported as the bootstrap median with 95% confidence intervals in parentheses, based on 100 bootstrap resamples of the held-out test set. Metrics are shown separately for each diagnostic class and overall performance. **Abbreviations:** hyperfunctional dysphonia (HD), laryngitis (L), non-pathological (NP), recurrent paresis (RP).

| Class | Precision | Recall | F1 | Accuracy |
| --- | --- | --- | --- | --- |
| NP | 0.864 (0.801–0.908) | 0.849 (0.788–0.908) | 0.857 (0.804–0.896) | — |
| RP | 0.579 (0.464–0.725) | 0.682 (0.526–0.805) | 0.626 (0.497–0.748) | — |
| HD | 0.468 (0.270–0.652) | 0.380 (0.216–0.523) | 0.416 (0.243–0.558) | — |
| L | 0.486 (0.343–0.689) | 0.556 (0.357–0.754) | 0.520 (0.351–0.657) | — |
| Overall | 0.600 (0.528–0.666) | 0.613 (0.536–0.676) | 0.601 (0.530–0.666) | 0.698 (0.646–0.756) |

**Table S4.9.** Performance of the Wav2Vec 2.0 model for voice disorder classification using additional synthetic data generated with Kartoffelbox. Values are reported as the bootstrap median with 95% confidence intervals in parentheses, based on 100 bootstrap resamples of the held-out test set. Metrics are shown separately for each diagnostic class and overall performance. **Abbreviations:** hyperfunctional dysphonia (HD), laryngitis (L), non-pathological (NP), recurrent paresis (RP).

| **Class** | **Precision** | **Recall** | **F1** | **Accuracy** |
| --- | --- | --- | --- | --- |
| NP | 0.901 (0.852–0.952) | 0.678 (0.601–0.746) | 0.773 (0.718–0.829) | — |
| RP | 0.349 (0.272–0.449) | 0.977 (0.933–1.000) | 0.514 (0.425–0.618) | — |
| HD | 0.400 (0.217–0.596) | 0.244 (0.130–0.372) | 0.305 (0.161–0.437) | — |
| L | 0.000 (0.000–0.000) | 0.000 (0.000–0.000) | 0.000 (0.000–0.000) | — |
| Overall | 0.416 (0.356–0.465) | 0.475 (0.434–0.510) | 0.400 (0.352–0.443) | 0.575 (0.521–0.629) |

**Table S4.10.** Performance of the WavLM model for voice disorder classification using additional synthetic data generated with Kartoffelbox. Values are reported as the bootstrap median with 95% confidence intervals in parentheses, based on 100 bootstrap resamples of the held-out test set. Metrics are shown separately for each diagnostic class and overall performance. **Abbreviations:** hyperfunctional dysphonia (HD), laryngitis (L), non-pathological (NP), recurrent paresis (RP).

| **Class** | **Precision** | **Recall** | **F1** | **Accuracy** |
| --- | --- | --- | --- | --- |
| NP | 0.891 (0.829–0.932) | 0.875 (0.807–0.935) | 0.881 (0.834–0.918) | — |
| RP | 0.655 (0.494–0.770) | 0.684 (0.500–0.803) | 0.663 (0.514–0.758) | — |
| HD | 0.525 (0.375–0.649) | 0.587 (0.423–0.768) | 0.555 (0.429–0.670) | — |
| L | 0.407 (0.245–0.596) | 0.321 (0.190–0.500) | 0.364 (0.222–0.525) | — |
| Overall | 0.617 (0.555–0.696) | 0.616 (0.561–0.699) | 0.614 (0.556–0.696) | 0.725 (0.669–0.782) |

**Table S4.11.** Performance of the equally weighted ensemble combining HuBERT baseline, Wav2Vec 2.0 trained with Kartoffelbox-np augmentation, and LSTM trained with Kartoffelbox-np augmentation for voice disorder classification. **Abbreviations:** hyperfunctional dysphonia (HD), laryngitis (L), non-pathological (NP), recurrent paresis (RP).

| **Class** | **Precision** | **Recall** | **F1** | **Accuracy** |
| --- | --- | --- | --- | --- |
| NP | 0.916 (0.870–0.950) | 0.831 (0.768–0.894) | 0.873 (0.824–0.907) | — |
| RP | 0.709 (0.564–0.848) | 0.616 (0.446–0.739) | 0.658 (0.518–0.762) | — |
| HD | 0.510 (0.371–0.632) | 0.674 (0.511–0.816) | 0.580 (0.453–0.678) | — |
| L | 0.487 (0.347–0.629) | 0.535 (0.354–0.745) | 0.517 (0.346–0.645) | — |
| Overall | 0.658 (0.575–0.720) | 0.672 (0.581–0.731) | 0.659 (0.571–0.715) | 0.733 (0.677–0.788) |

**Table S4.12.** Performance of the Learnable Fusion (LF) approach for voice disorder classification. Values are reported as the bootstrap median with 95% confidence intervals in parentheses, based on 100 bootstrap resamples of the held-out test set. Metrics are shown separately for each diagnostic class and overall performance. **Abbreviations:** hyperfunctional dysphonia (HD), laryngitis (L), non-pathological (NP), recurrent paresis (RP).

| **Class** | **Precision** | **Recall** | **F1** | **Accuracy** |
| --- | --- | --- | --- | --- |
| NP | 0.894 (0.830–0.937) | 0.840 (0.781–0.916) | 0.867 (0.820–0.909) | — |
| RP | 0.750 (0.618–0.889) | 0.579 (0.420–0.738) | 0.644 (0.516–0.787) | — |
| HD | 0.500 (0.329–0.632) | 0.582 (0.412–0.750) | 0.538 (0.397–0.651) | — |
| L | 0.449 (0.300–0.608) | 0.596 (0.393–0.797) | 0.516 (0.341–0.657) | — |
| Overall | 0.648 (0.571–0.711) | 0.649 (0.563–0.723) | 0.640 (0.561–0.707) | 0.721 (0.669–0.775) |

**Table S4.13.** Performance of the Learnable Fusion with Sex Embedding (LFS) approach for voice disorder classification. Values are reported as the bootstrap median with 95% confidence intervals in parentheses, based on 100 bootstrap resamples of the held-out test set. Metrics are shown separately for each diagnostic class and overall performance. **Abbreviations:** hyperfunctional dysphonia (HD), laryngitis (L), non-pathological (NP), recurrent paresis (RP).

| Original SVD label | English equivalent | Frequency |
| --- | --- | --- |
| Dysarthrophonie | Dysarthrophonia | 19 |
| Rhinophonie mixta | Mixed rhinolalia | 1 |
| Diplophonie | Diplophonia | 5 |
| Monochorditis | Monochorditis | 3 |
| Dysplastischer Kehlkopf | Dysplastic larynx | 1 |
| Frontolaterale Teilresektion | Frontolateral partial resection | 35 |
| Hypopharynxtumor | Hypopharyngeal tumor | 6 |
| N. laryngeus superior Neuralgie | Superior laryngeal nerve neuralgia | 3 |
| Taschenfaltenstimme | Ventricular fold voice | 11 |
| Dish-Syndrom | DISH syndrome | 1 |
| Balbuties | Stuttering | 20 |
| Juvenile Dysphonie | Juvenile dysphonia | 1 |
| Phonasthenie | Phonasthenia | 10 |
| Chordektomie | Cordectomy | 59 |
| Reinke Ödem | Reinke edema | 68 |
| Dysphonie | Dysphonia | 101 |
| Zentral-laryngale Bewegungsstörung | Central laryngeal movement disorder | 14 |
| N. laryngeus superior Läsion | Superior laryngeal nerve lesion | 3 |
| Funktionelle Dysphonie | Functional dysphonia | 112 |
| Gesangsstimme | Singing voice disorder | 17 |
| Psychogene Dysphonie | Psychogenic dysphonia | 91 |
| Non-fluency-Syndrom | Non-fluency syndrome | 2 |
| Vox senilis | Vox senilis | 40 |
| Rhinophonie aperta | Open rhinolalia | 18 |
| Mutationsfistelstimme | Mutational falsetto voice | 10 |
| Spasmodische Dysphonie | Spasmodic dysphonia | 64 |
| Phonationsknötchen | Vocal fold nodules | 18 |
| Hypotone Dysphonie | Hypotonic dysphonia | 5 |
| Kontaktpachydermie | Contact pachydermia | 71 |
| Leukoplakie | Leukoplakia | 41 |
| Dysodie | Dysodia | 56 |
| Sängerstimme | Singer's voice disorder | 2 |
| Velopharyngoplastik | Velopharyngoplasty | 2 |
| Stimmlippenkarzinom | Vocal fold carcinoma | 22 |
| Stimmlippenpolyp | Vocal fold polyp | 45 |
| Laryngitis | Laryngitis | 140 |
| Hyperfunktionelle Dysphonie | Hyperfunctional dysphonia | 213 |
| Fibrom | Fibroma | 2 |
| Rekurrensparese | Recurrent laryngeal nerve paresis | 213 |
| Bulbärparalyse | Bulbar palsy | 2 |
| Synechie | Synechia | 2 |
| Intubationsgranulom | Intubation granuloma | 4 |
| GERD | Gastroesophageal reflux disease | 3 |
| Hypofunktionelle Dysphonie | Hypofunctional dysphonia | 16 |
| Rhinophonie clausa | Closed rhinolalia | 1 |
| Orofaciale Dyspraxie | Orofacial dyspraxia | 1 |
| Epiglottiskarzinom | Epiglottic carcinoma | 1 |
| Kehlkopftumor | Laryngeal tumor | 5 |
| Psychogene Aphonie | Psychogenic aphonia | 1 |
| Sigmatismus | Sigmatism | 4 |
| Aryluxation | Arytenoid luxation | 6 |
| Laryngozele | Laryngocele | 3 |
| Amyotrophe Lateralsklerose | Amyotrophic lateral sclerosis | 2 |
| Internusschwäche | Internal weakness | 1 |
| Valleculacyste | Vallecular cyst | 1 |
| Psychogene Mikrophonie | Psychogenic microphonia | 1 |
| Chondrom | Chondroma | 1 |
| Cyste | Cyst | 6 |
| Taschenfaltenhyperplasie | Ventricular fold hyperplasia | 2 |
| Morbus Down | Down syndrome | 1 |
| Intubationsschaden | Intubation injury | 3 |
| Mutatio | Voice mutation disorder | 2 |
| Carcinoma in situ | Carcinoma in situ | 1 |
| Mediale Halscyste | Median neck cyst | 1 |
| Mesopharynxtumor | Mesopharyngeal tumor | 1 |
| Morbus Parkinson | Parkinson disease | 1 |
| Hyperasthenie | Hyperasthenia | 1 |
| Dysplastische Dysphonie | Dysplastic dysphonia | 1 |
| Granulom | Granuloma | 2 |
| Poltersyndrom | Cluttering | 3 |
| Papillom | Papilloma | 1 |
| Not pathologic | Non-pathological | 869 |

Table S5. Distribution and frequency of diagnostic-label assignments in the Saarbruecker Voice Database. Original SVD label strings are retained for traceability to the source database, and English equivalents are provided in the second column. Frequencies reflect diagnostic-label assignments, with multiple diagnoses possible for individual pathological speakers. The table comprises 1,626 pathological diagnostic-label assignments and 869 non-pathological entries (2,495 total assignments/counts) across 2,225 speaker/recording-session metadata entries.

| **Parameter** | **Value** |
| --- | --- |
| Model | XTTS v2 (Coqui TTS) |
| Language | German (de) |
| Speaker conditioning | Corresponding phrase recording |
| Temperature | 0.85 |
| Top-k | 50 |
| Top-p | 0.85 |
| Repetition penalty | 2.0 |
| Output sample rate | 24,000 Hz |

**Table S6.** Generation parameters used for XTTS-based synthetic speech generation. German-language synthesis was enforced using the XTTS language parameter (de). Speaker characteristics were conditioned using the corresponding phrase recording from the Saarbruecker Voice Database.

| **Comparison** | **Class** | **Metric** | Median Δ (95% bootstrap percentile CI) |
| --- | --- | --- | --- |
| HuBERT XTTS-np − Original | Overall | Recall | 0.0366 (0.0186–0.0657) |
| HuBERT XTTS-np − Original | Overall | Specificity | −0.0731 (−0.1249–−0.0278) |
| LSTM XTTS-np − Original | Overall | Recall | −0.0335 (−0.0772–0.0074) |
| LSTM XTTS-np − Original | Overall | Specificity | 0.0303 (−0.0080–0.0949) |
| LF − LFS | Overall | Accuracy | 0.0125 (−0.0252–0.0502) |
| LF − LFS | Overall | F1-score | 0.0132 (−0.0407–0.0679) |
| LF − LFS | Hyperfunctional dysphonia | Recall | 0.0930 (0.0000–0.2024) |
| LF − LFS | Laryngitis | F1-score | 0.0065 (−0.0930–0.1121) |
| LF − Equal-weight ensemble | Overall | F1-score | 0.0402 (−0.0191–0.0989) |
| LFS − Equal-weight ensemble | Overall | F1-score | 0.0266 (−0.0445–0.0942) |
| LF − HuBERT + Kartoffelbox | Laryngitis | F1-score | −0.0004 (−0.1330–0.1144) |
| LF − Wav2Vec 2.0 + Kartoffelbox | Laryngitis | F1-score | −0.0189 (−0.1091–0.1246) |
| LF − HuBERT Kartoffelbox-np | VDR Overall | Accuracy | 0.0101 (−0.0226–0.0403) |
| LF − HuBERT Kartoffelbox-np | VDR Overall | F1-score | 0.0098 (−0.0249–0.0428) |

Table S7. Paired bootstrap differences in performance between the evaluated model configurations. Differences (Δ) are expressed as the first model or approach minus the second and are summarized as median differences with 95% bootstrap percentile confidence intervals across 100 aligned bootstrap iterations. Abbreviations: hyperfunctional dysphonia (HD), Learnable Fusion (LF), Learnable Fusion with Sex Embedding (LFS), Long Short-Term Memory (LSTM), Voice Disorder Classification (VDC), Voice Disorder Recognition (VDR), XTTS-based augmentation (XTTS).
